# A quantum dots and superparamagnetic nanoparticle-based method for the detection of HPV DNA

**DOI:** 10.1186/1556-276X-6-461

**Published:** 2011-07-20

**Authors:** Wang Yu-Hong, Chen Rui, Li Ding

**Affiliations:** 1 Emergency Department, General Hospital of Beijing Military Area of PLA, Beijing 100700, China; 2 The Department of Blood Transfusion, Xijing Hospital, The Fourth Military Medical University, Xian 710032, China; 3 Center of Biological Diagnosis and Therapy, No. 261 Hospital of PLA, Beijing 100094, China

**Keywords:** HPV, DNA, quantum dots, superparamagnetic nanoparticles, hybridization, cervical cancer

## Abstract

**Background:**

The recent advance in nanomaterial research field prompts the development of diagnostics of infectious diseases greatly. Many nanomaterials have been developed and applied to molecular diagnostics in labs. At present, the diagnostic test of human papillomavirus (HPV) relies exclusively on molecular test. Hereon, we report a rapid and facile quantum dots (QDs) and superparamagnetic nanoparticle-based hybridization assay for the detection of (HPV) 16 infections which combines the merits of superparamagnetic nanoparticles and QDs and wholly differs from a conventional hybridization assay at that the reaction occurs at homogeneous solution, and total time for detection is no more than 1 h.

**Methods:**

The probes were labeled with superparamagnetic nanoparticles and QDs. Sixty cervical swab samples were used to perform a hybridization assay with these probes, and the results were compared with type-specific polymerase chain reaction (PCR) method.

**Results:**

The statistic analysis suggests that there is no significant difference between these two methods. Furthermore, this method is much quicker and easier than the type-specific PCR method.

**Conclusion:**

This study has successfully validated the clinical performance of our hybridization assay. The advantages in the time of detection and ease of process endow this method with great potential in clinical usage, especially mass epidemiological screening.

## Introduction

Human papillomavirus (HPV) is a small non-enveloped DNA virus that merely infects human squamous epithelial cells. Its genome is a double-stranded circular DNA molecule of 8,000 base pairs (bp) which is divided into three parts, including a segment of about 4,000 bp that encodes proteins mainly involved in viral DNA replication and cell transformation, a segment of about 3,000 bp that encodes the structural proteins of the virus particles as well as a segment of about 1,000 bp that contains the origin of viral DNA replication and transcriptional regulatory elements [1,2]. HPVs can cause a large spectrum of epithelial lesions, primarily benign hyperplasia with low malignant potential such as warts, papillomas, and so forth. Based on epidemiological and molecular evidence, HPV types 16 and 18 were recognized as the high-risk types that were carcinogenic in humans [2,3]. HPV-16 accounts for approximately 50% of all cervical cancers, while HPV-18 is the next most common type and typically is found in from 15% to 20% of squamous cell cancers and in a greater proportion of adenocarcinomas [2-6]. However, cervical cancer is a highly preventable disease when early screening programs are employed that facilitate the detection and treatment of precancerous lesions. Assisted by early detection, the 5-year survival rate for the earliest stage of invasive cervical cancer can be fairly high [7,8].

In recent years, various nanomaterials have been applied to the field of molecular diagnostics [9,10]. Quantum dots (QDs), one of these nanomaterials, are nearly spherical semiconductor particles with diameters from 2 to 10 nm, comprising 200 to 10,000 atoms. QDs have size-controlled luminescence functions, which mean the same material with variable sizes can exhibit different colors under the excitation of an appropriate wavelength; broad absorption spectra; and narrow emission spectra, which mean simultaneous excitation of different colored QDs by a single wavelength [11,12]. In addition, QDs are extremely photostable and highly resistant to photobleaching, which has been reported to be more photostable than a number of organic dyes, including the most stable organic dye, Alexa 488 [13,14]. With their rapid progress, various QDs-bioconjugates have been developed for imaging, labeling, and sensing [15]. Manipulable superparamagnetic nanoparticle through contrived magnetic field is another outstanding nanomaterial, which has been applied to magnetic resonance imaging contrast enhancement, immunoassay, hyperthermia, magnetic drug delivery, magnetofection, cell separation, or cell labeling [16]. Especially in biological separation and diagnosis, the superparamagnetic nanoparticle has a unique advantage over others.

Herein, we report a novel detection method of HPV DNA combining the advantages of QDs and manipulability of superparamagnetic nanoparticles and validate it clinically.

## Methods

### Collection of samples

One hundred sixty cervical swab samples were collected from outpatients at our department, and the written informed consent was obtained. Ten HPV-16-negative and ten HPV-16-positive human DNA samples were kept in the clinical laboratory of our department. QIAamp^® ^DNA Blood Mini Kits (Qiagen) were used to extract DNA according to the manufacturer's protocol. All DNA samples were eluted with the same volume and then frozen in -70°C until further analysis after quantitated with UV spectrometer (Beckman Coulter, Inc., Beijing, People's Republic of China).

### Preparation of CdTe QD-labeled DNA probes

The QD-labeled DNA probes were synthesized according to MY Gao and Dai Zhao [17,18]. In brief, firstly, tellurium powder and NaBH_4 _was added into a 100-mL flask with 50 mL of Milli-Q water. The reaction was implemented in room temperature with N_2 _protection and lasted until the Tellurium powder disappeared in the flask. Secondly, 86.6 mg of CdCl2 and 79.22 μL of 3-mercaptopropionic acid were dissolved in a three-necked flask with 297 mL of Milli-Q water under N_2 _protection. One molar NaOH solution was used to adjust the pH of the mixture to 9.1 under stirring. The NaHTe solution prepared in the first step was added to the reaction mixture under N_2 _protection. The resultant mixture was stirred for about 20 min and then boiled at 100°C. The reflux time to get the CdTe QDs was 1 h. X-Ray diffraction (XRD) was used to confirm the crystalline phase of QDs. Four milliliter of CdTe QDs, approximately 100 μg of DNA oligonucleotide second probe described by Lee *et al*. [19] (Table 1) and 1-ethyl-3-(3-dimethylaminopropyl) carbodiimide hydrochloride (EDAC) amounting to ten times the mole of DNA, were mixed in 0.05 M Tris-HCl and 0.02 M NaCl buffer (pH 7.2) under room temperature. The resultant product was CdTe QD-labeled probe, and excessive oligonucleotide probes were removed by dialysis against a pH 7.0 PBS buffer using a cellulose-acetate membrane. The emission spectrum of resultant QD-labeled probes was characterized by LS 55 luminescence spectrometer (Perkin-Elmer, Beijing, China). Sodium dodecyl sulfate polyacrylamide gel electrophoresis (SDS-PAGE) was used to verify the conjugation of QDs and probes.

**Table 1:** Hybridization probes and type-specific PCR primers

	Sequence
Capture probe	5-GAGGAGGATGAAATAGATGGTCCAGCTGGACAAGCAGAACCGGACAGAGCCCATTACAATATTGTAACCTTTTGTTGCAAGTGTGACTCTACGCTTCGGT-3
Secondary probe	5-GGAGCGACCCAGAAAGTTACCACAGTTATGCACAGAGCTGCAAACAACTA-3
Type-specific PCR upper primer	TGT GCT GCC ATA TCT ACT TCA GAA ACT AC
Type-specific PCR lower primer	TAG ACC AAA ATT CCA GTC CTC CAA A

### Preparation of superparamagnetic nanoparticle

The superparamagnetic nanoparticles were synthesized according to Nagao *et al*. with slight modification [20]. Briefly, 5 mL of 2-M FeCl_2 _and 20 mL of 1-M FeCl_3 _were mixed in 212 mL of Milli-Q water that had been bubbled with nitrogen for 30 min. Fe_3_O_4 _nanoparticles were chemically co-precipitated by adding 12 mL of NH_3 _solution at room temperature under continuous mixing and washed four times in water and several times in ethanol. During washing, the superparamagnetic Fe_3_O_4 _nanoparticles were separated with a NdFeB magnet, and the particles were finally dried in a vacuum oven at 70°C. The transmission electron microscopy (JEOL, Tokyo, Japan) was used to characterize the size of the magnetic nanoparticles. XRD was used to confirm the crystalline phase of superparamagnetic nanoparticles.

### Modification and coupling of superparamagnetic nanoparticle

3-Aminopropyl-trimethoxysilane (APTMS) modification and coupling process of superparamagnetic nanoparticles were prepared according to the method described by Kouassi *et al*. [21]. One gram of Fe_3_O_4 _nanoparticles were washed with methanol and Milli-Q water and then added to 10 mL of 3 mM APTMS in a toluene/methanol with a ratio of 1:1 in volume in a three-necked flask with a condenser and temperature controller protected by N_2 _at 80°C for 20 h under vigorous stirring. Amino group-modified Fe_3_O_4 _nanoparticles were separated by a NdFeB magnet and washed several times with methanol and Milli-Q water alternately and then dried at 50°C in a vacuum oven. Approximately 50 mg of APTMS-modified Fe_3_O_4 _nanoparticles was added into 10 mL of 0.05 mg/mL of EDAC and sonicated for 25 min at 4°C. After being separated with a NdFeB magnet, 50 nmol of streptavidin in a phosphate buffer solution was added. The resultant mixture was sonicated for 1 h, and the particles coupled with streptavidin were magnetically extracted. SDS-PAGE was used to verify the conjugation of the superparamagnetic nanoparticles and probes.

### Determine of cutoff value and validation of QDs and superparamagnetic nanoparticle-based hybridization

**Figure 1:**
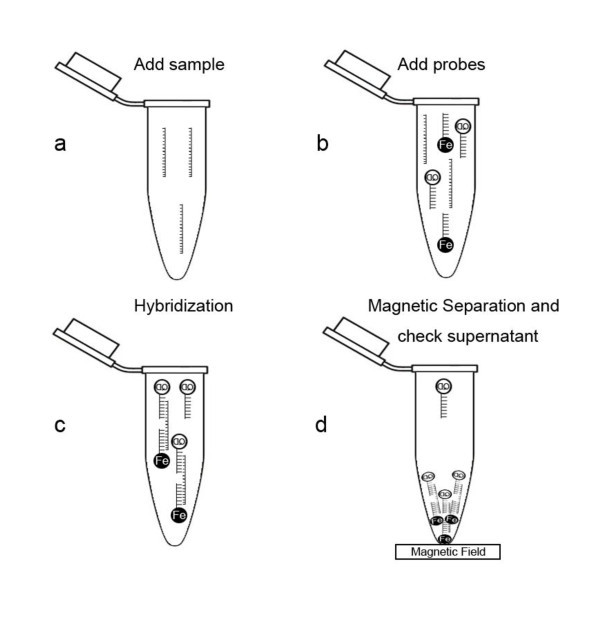
**The rationale of QDs and superparamagnetic nanoparticle-based hybridization**.

Ten HPV-16-negative human DNA samples were used to determine the cutoff value of QDs and superparamagnetic nanoparticle-based hybridization. The detection procedure was described in detail in the next section (Figure 1). The cutoff value was defined as the mean fluorescence intensity of HPV-16-negative human DNA samples minus double standard deviations (CV). A result under cutoff value in succedent detection was determined as a positive result. The ten HPV-16-positive samples were used to validate our hybridization assay on the basis of the cutoff value.

### Detection of HPV-16 with QDs and superparamagnetic nanoparticle-based hybridization

The rationale of QDs and superparamagnetic nanoparticle-based hybridization is illustrated in Figure 1. A 0.05-μg biotin-labeled capture probes and QD-labeled detective probes described by Lee *et al*. [19] (Table 1) were mixed adequately with 2 μL of DNA samples in a volume with a total of 100-μL-long oligo hybridization solution (Corning Incorporated, Shanghai, China) and predenatured at 95°C for 10 min, then 55°C for 30 min. The particles coupled with streptavidin were added into the hybridization mixtures and incubated at 37°C for 10 min and enriched in the bottom of the tube with a NdFeB magnet. A 20-μL supernatant was taken to measure relative fluorescence intensity by LS 55 luminescence spectrometer (Perkin-Elmer, Beijing, China).

### Detection of HPV16 with type-specific PCR

The 160 DNA samples were also analyzed with type-specific polymerase chain reaction (PCR) according to Lin *et al*. [22] (Table 1). The PCR reaction system consisted of 3 μL DNA sample, 15 mM Tris-HCl (pH 8.0), 2.5 mM MgCl_2_, 50 mM KCl, 0.25 mM dNTPs, 10 μM upper and lower primers, and 0.5 U of Hot-Start Taq DNA polymerase (Takara, Otsu, Shiga, Japan). The PCR reaction mixture was preheated for 5 min at 94°C, followed by 45 cycles of 30 s at 94°C, 30 s at 59°C, 30 s at 72°C, and a final extension of 5 min at 72°C. A no-template reaction was implemented in each assay as negative control, and each sample was performed in triplicate. PCR products were analyzed in 1% agarose gel electrophoresis.

### Statistical analysis

The comparison between QDs and superparamagnetic nanoparticle-based hybridization and type-specific PCR was analysized by the Statistics Package for Social Sciences (SPSS) software. A *p *value above 0.05 was considered that there was no significant difference between the two methods.

## Results

### Characterization of quantum dots

**Figure 2:**
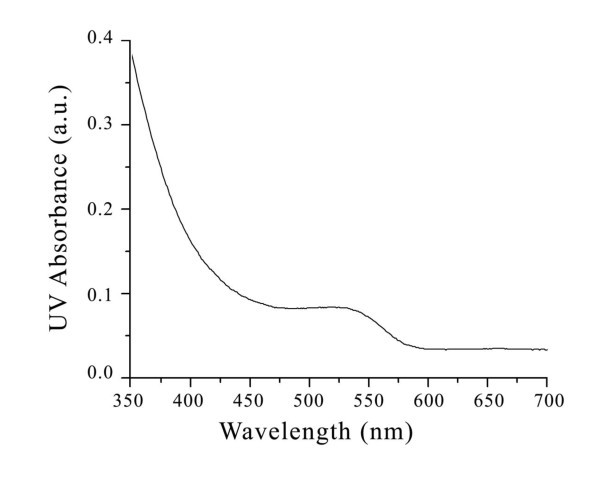
**The UV absorbance spectrum of QDs**.

**Figure 3:**
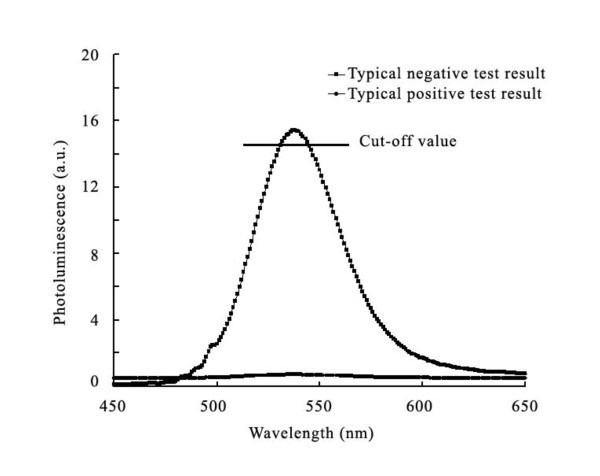
**Fluorescent spectrum of QDs**.

**Figure 4:**
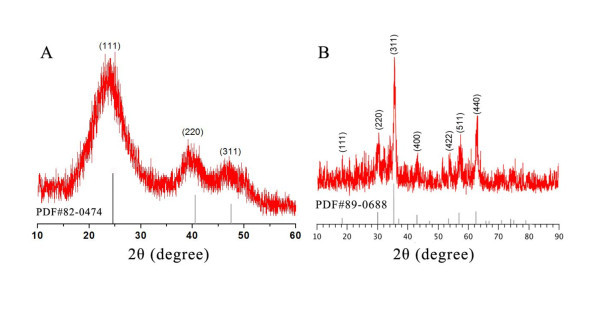
**X-ray diffraction analysis of QDs and superparamagnetic nanoparticles**.

**Figure 5:**
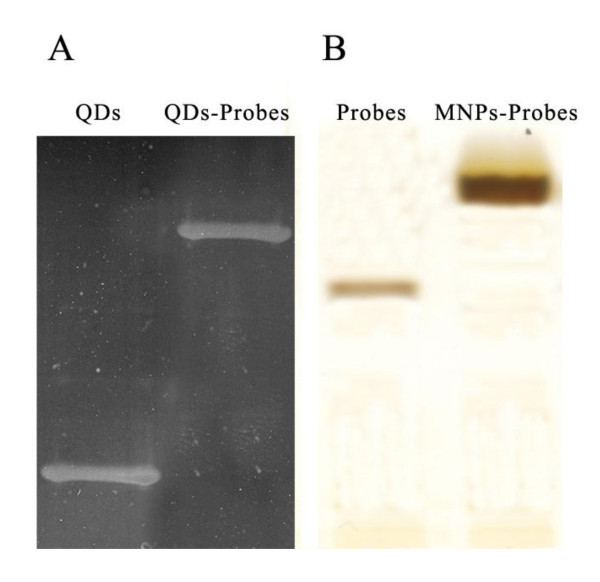
**SDS-PAGE results of QDs and superparamagnetic nanoparticles**.

The as-prepared quantum dots are red solution. According to the absorbance spectrum and emission spectrum measured by UV spectrophotometer and luminescence spectrometer, they could be excited effectively under ultraviolet band, and their maximum emission peak is about 530 nm, which means the resultant quantum dots is fluorescence-active and could be used as a fluorescent probe (Figures 2, 3). The X-Ray diffraction analysis indicates that the as-prepared QDs exhibit a zinc blende cubic structure (Figure 4A). The position and relative intensity of most peaks match well with standard CdTe powder diffraction data (JCPDS82-0474). The SDS-PAGE results under UV lamp indicate that probes have been conjugated to QDs (Figure 5A).

### Characterization of superparamagnetic nanoparticles

**Figure 6:**
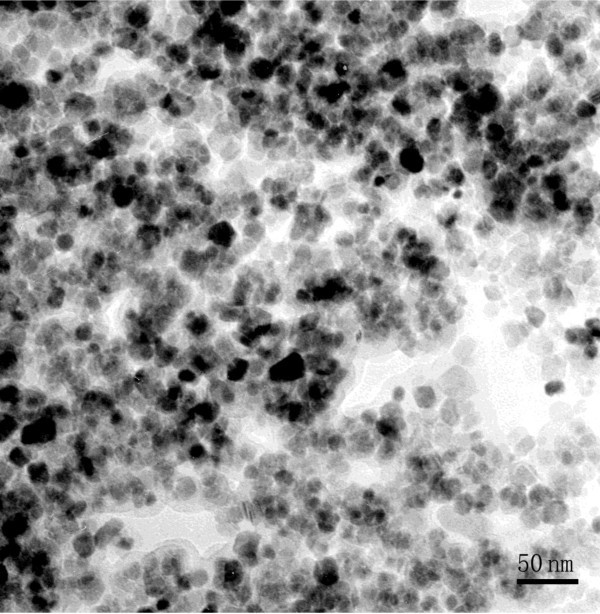
**TEM characterization of superparamagnetic Fe_3_O_4 _nanoparticles**.

**Figure 7:**
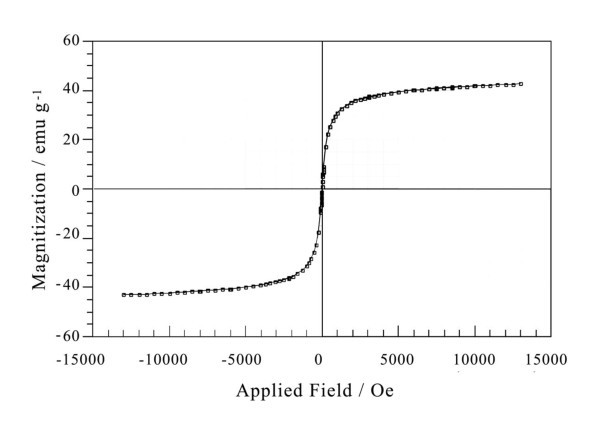
**VSM result of as-synthesized superparamagnetic nanoparticles**.

To demonstrate the formation of superparamagnetic nanoparticles, the as-prepared Fe_3_O_4 _solution was dropped on the copper grid coated with carbon film and characterized by transmission electron microscopy (JEOL, Tokyo, Japan. As seen in Figure 6, the size of Fe_3_O_4 _nanoparticles is about 20 nm. The power XRD pattern also shows that the as-prepared magnetite nanoclusters have an inverse spinel type structure (Figure 4B). The position and relative intensity of most peaks match well with standard Fe_3_O_4 _powder diffraction data (JCPDS89-0688), indicating that the magnetite nanocrystals in nanoclusters are crystalline. In addition, the nanoparticles could be enriched in 2 min by a NdFeB magnet, which means they have good magnetic property. After the removal of external magnetic field, these particles could be easily dispersed, suggesting their paramagnetism. The vibrating sample magnetometer (VSM) results of as-synthesized superparamagnetic nanoparticles indicate that they exhibit superparamagnetic behavior with a saturation moment of about 42.5 emu/g at 300 K, as shown in Figure 7. The SDS-PAGE results under silver staining indicate that probes have been conjugated to superparamagnetic nanoparticles (Figure 5B).

### The cutoff value of QDs and superparamagnetic nanoparticle-based hybridization

Ten HPV-16-negtive samples were repeated three times with the abovementioned method; the means were used to determine the cutoff value. According to the data, the cutoff value of this assay was defined as 14.5, any result under 14.5 from the 160 DNA samples was considered as positive one (Figure 3). Based on this cutoff value, all of the ten HPV-16-positve DNA samples were determined as positive results.

### Comparison of QDs and superparamagnetic nanoparticle-based hybridization with type-specific PCR

The 160 outpatients' DNA samples were checked with QDs and superparamagnetic nanoparticle-based hybridization and type-specific PCR. The results were analyzed with the SPSS software. According to our assay, the infectious rate of HPV 16 in these female outpatients is about 8.1% (13/160) by hybridization method and about 6.9% (11/160) by type-specific PCR method. All samples were detected by DNA sequencing, and the two samples with controversial results were confirmed positive. However, no significant difference was seen between the two methods for analysis of the paired *χ*^2 ^test (Table 2).

**Table 2:** Comparison between QDs and superparamagnetic nanoparticle-based hybridization and type-specific PCR

Hybridization	Type-specific PCR		Sum
		
	Positive	Negative	
Positive	11	2	13
Negative	0	147	147
Sum	11	149	160

*χ^2 ^*= 0.50; *p *> 0.05

## Discussion

In this paper, we have successfully developed a novel and facile hybridization for the qualitative detection of HPV-16 in cervical swab samples. Compared with type-specific PCR, the greatest advantages of our QDs and superparamagnetic nanoparticle-based hybridization consists in the time of detection and ease of process. Generally speaking, type-specific PCR for detection of HPV-16 DNA takes a skillful laboratory assistant about 4 h, while our hybridization assays only need no more than 1 h. In addition, a typical type-specific PCR assay consists of the extraction of DNA of cervical swab samples, PCR reaction and nucleic acid agarose gel electrophoresis and staining of ethidium bromide, while our hybridization assay method only require extraction of DNA of the samples and simple incubation as well as magnetic separation, which has a good acceptability for any average lab assistant.

With the increasing interest in the development of diverse nanomaterials, many researchers all over the world are pushing the envelope to expand the application of those versatile materials in the field of medicine. Up to the present, numerous nanomaterials have been applied to diagnose infectious diseases such as human immunodeficiency virus, respiratory syncytial virus, hepatitis B virus, hepatitis C virus (HCV), hepatitis E virus, herpes simplex virus, and so forth [23-28]. Surely, nanotechnology brings new opportunities in diagnostics which allows for the diagnosis of infectious diseases in a sensitive, specific, and rapid format at lower costs than current in-use technologies. As declared by Jain KK, applications of nanotechnology are beginning to show an impact on the practice of conventional medicine; it is bound to continue as hotspot of research for next several decades [28].

In conclusion, we showed a rapid and facile hybridization method for the qualitative detection of HPV-16 DNA in cervical swab samples and successfully validated it in 160 clinical samples. It differs from conventional hybridization assays in such a way that the reaction occurs at homogeneous solution and that of conventional hybridization assay bases on the solid supporter such as polyvinylidene fluoride membrane or nitrocellulose membrane. Therefore, this method has great potential in clinical usage, especially mass epidemiological screening.

## Competing interests

The authors declare that they have no competing interests.

## Authors' contributions

WYH carried out the molecular diagnostic study. CR participated in the collection of clinical samples and part of molecular diagnostic study. LD conceived of the study, and participated in its design, performed the preparation of nanomaterials and the statistical analysis. All authors read and approved the final manuscript.
